# Novel *de novo* frameshift variant in the *ASXL3* gene in a child with microcephaly and global developmental delay

**DOI:** 10.3892/mmr.2019.10303

**Published:** 2019-05-27

**Authors:** Marketa Wayhelova, Jan Oppelt, Jan Smetana, Eva Hladilkova, Hana Filkova, Eva Makaturova, Petra Nikolova, Rastislav Beharka, Renata Gaillyova, Petr Kuglik

**Affiliations:** 1 Institute of Experimental Biology, Faculty of Science, Masaryk University, 611 37 Brno, Czech Republic; 2 Department of Medical Genetics, University Hospital Brno, 625 00 Brno, Czech Republic; 3 CEITEC-Central European Institute of Technology, Masaryk University, 625 00 Brno, Czech Republic; 4 National Centre for Biomolecular Research, Faculty of Science, Masaryk University, 625 00 Brno, Czech Republic

**Keywords:** developmental delay, Bainbridge-Ropers syndrome, array-CGH, next-generation sequencing, additional sex-combs like 3 gene

## Abstract

*De novo* sequence variants, including truncating and splicing variants, in the additional sex-combs like 3 gene (*ASXL3*) have been described as the cause of Bainbridge-Ropers syndrome (BRS). This pathology is characterized by delayed psychomotor development, severe intellectual disability, growth delay, hypotonia and facial dimorphism. The present study reports a case of a girl (born in 2013) with severe global developmental delay, central hypotonia, microcephaly and poor speech. The proband was examined using a multi-step molecular diagnostics algorithm, including karyotype and array-comparative genomic hybridization analysis, with negative results. Therefore, the proband and her unaffected parents were enrolled for a pilot study using targeted next-generation sequencing technology (NGS) with gene panel ClearSeq Inherited Disease^XT^ and subsequent validation by Sanger sequencing. A novel *de novo* heterozygous frameshift variant in the *ASXL3* gene (c.3006delT, p.R1004Efs*21), predicted to result in a premature termination codon, was identified. In conclusion, the present study demonstrated that targeted NGS using a suitable, gene-rich panel may provide a conclusive molecular genetics diagnosis in children with severe global developmental delays.

## Introduction

The advent of the next-generation sequencing (NGS) technology has revolutionized the current approaches in molecular diagnostics of individuals with severe intellectual disabilities (ID), developmental delay (DD), autism spectrum disorders (ASD) and multiple congenital abnormalities (MCA). Recently, >1,000 genes have been identified as the cause of ID and other neurodevelopmental disorders (1).

A previous study showed that >30% of cases of idiopathic ID and 13% of cases of severe ID (IQ<50) can be explained with a specific molecular diagnosis using a targeted NGS approach (2). Moreover, the current molecular diagnostic method uses whole-exome sequencing (WES), which is a highly powerful and cost-effective part of the first-tier standard diagnostic approach, with a diagnostic yield of up to 55% (3).

Bainbridge-Ropers syndrome (BRS; OMIM #615485) is a rare congenital disorder characterized by delayed neuronal, motor and growth development, severe ID accompanied by absent or poor speech, muscular hypotonia, feeding difficulties and facial dimorphism (4). It was first described by Bainbridge *et al* (5), who identified rare de novo truncating sequence variants in the additional sex-combs like 3 (*ASXL3*) gene in a group of 4 unrelated affected children presenting similar phenotypic features. The pathogenic sequence variants in the *ASXL3* gene lead to the reduced expression of the gene. The haploinsuffiency of the *ASXL3* gene with high penetrance is the most likely the underlying and causative mechanism of the disease (6).

In the present study, the case of a child (female, born in 2013) with global developmental delay, central hypotonia, microcephaly and poor speech is described. She was examined using a multi-step molecular diagnostics algorithm, including karyotype and array-comparative genomic hybridization (CGH) analyses. However, no pathogenic chromosomal rearrangements or copy-number variants (CNVs) that could explain the phenotype of the patient were identified. Therefore, the patient and her healthy parents took part in a pilot study using targeted NGS with a commercially available gene-rich panel. This panel contained 2,742 genes catalogued in the Online Mendelian Inheritance in Man (OMIM) database whose pathogenic sequence variants are associated with human inherited diseases. The results of this approach were subsequently validated using consecutive Sanger sequencing.

## Materials and methods

### 

#### Clinical characteristics of the patient

The proband was born from the second pregnancy of healthy, unrelated parents (mother and father both born in 1981). The mother has history of reproductive problems and the couple attempted to have children for 2 years (from age 28 to 30 years). After hormonal stimulation therapy, the mother conceived spontaneously. The proband was delivered by breech birth after Cesarean section as a dizygotic twin at 34 weeks of gestation (2,160 g/45 cm reaching Apgar score 10-10-10, and the brother 2,460 g/45 cm). The prenatal ultrasound and biochemical screening did not show any apparent abnormalities. After delivery, the proband experienced neonatal icterus followed by 2-day-long phototherapy, and developed a poor sucking reflex. The proband was transferred to specialized neonatal unit, where she spent 18 days in the neonatal incubator. She was vaccinated according to the recommended vaccination schedule.

The proband was breast-fed up 7 months of the age, with persisting feeding difficulties. Since the neonatal period, she had been diagnosed with central hypotonia, delayed psychomotor development and microcephaly, with an occipitofrontal circumference (OFC) of 34.5 cm at 7 weeks of age. To improve motor development, the proband was stimulated using Vojta therapy (7,8). At the age of 6 months, a brain ultrasound examination did not uncover any abnormalities. The proband started to roll over at the age of 13 months, as well as crawling and standing on four limbs during the following month, without forward motion. Brain magnetic resonance imaging (MRI) and electroencephalography did not show any abnormalities at the age of 18 months, and the proband had an OFC of 42.8 cm. At present, the proband exhibits mild facial dimorphism including convergent strabismus, palate malformations and severely delayed milestones in physiological and intellectual developmental stages. The proband does not currently display coordinated gross motor skills (walking) due to the persistent central hypotonia and feet malformations. Due to the hypotonia, her head often leans backwards. The proband does not speak at present, only vocalizes. At the age of 9 months, she developed short-term recurrent infections of the upper respiratory tract, which were treated with antibiotics. Regarding skeletal and skin abnormalities, the proband has a 4-finger line on the right palm, and the first and third toes are crossed under the second toe on both feet. Some autistic traits, including unprovoked laughter, have been identified, but social contacts have started to develop. The proband is under multidisciplinary specialized medical supervision and is undergoing rehabilitative therapy. In summary, her psychomotor development corresponds to that of a 1-year-old child. Her dizygotic twin brother is healthy, and her older brother (born in 2012) suffers from a cleft lip and mild facial dimorphism (hypertelorism and epicanthus).

Table I provides an overview of the phenotypic features of the proband and a brief summary of selected previously reported BRS patients with the *ASXL3* gene pathogenic variants.

The proband was diagnosed at the Department of Medical Genetics (University Hospital Brno) at the age of 18 months in April 2015. The patient's parents provided written informed consent. Peripheral blood samples were collected in sterile heparinized tubes for cytogenetic analysis. Genomic DNA samples were obtained from 1 ml peripheral blood in EDTA, according to the standard DNA isolation process using the MagNaPure system (Roche Diagnostics, Basel, Switzerland). Quality and quantity were checked using a NanoDrop^®^ ND-1000 (Thermo Fisher Scientific, Inc., Waltham, MA, USA) and Qubit^®^ 1.0 (Thermo Fisher Scientific, Inc.).

#### G-banded karyotype analysis and array-CGH technique

Cytogenetic analysis of the karyotype was performed using a standard G-banding procedure as previously described (9,10). Whole-genomic screening of unbalanced chromosomal rearrangements by array-CGH was performed using SurePrint G3 CGH Microarray 4×180 K (Agilent Technologies, Inc., Santa Clara, CA, USA), following the manufacturer's recommendations. The patient's DNA sample was matched with Human Genomic DNA, Female reference (Promega Corporation, Madison, WI, USA). The microarray slide was scanned with a DNA Microarray Scanner (Agilent Technologies, Inc.). Data were obtained using Agilent Feature Extraction software, version 12.0.2.2, and visualized using Agilent Genomic Workbench Software, version 7.0.4.0 (both from Agilent Technologies, Inc.). Structural CNVs were detected using the ADM-2 algorithm (11) with the following filters: >5 neighboring probes in genomic region; minimal size of 200 kb in region; and minimal absolute average log ratio of 0.25 as cut-off. All genomic positions were estimated on the human reference sequence GRCh37/hg19. Microarray data are available in the Array Express database (https://www.ebi.ac.uk/arrayexpress/) under the accession number E-MTAB-7027.

#### Targeted NGS

High quality genomic DNA was used for the library preparation. A total of 200 ng DNA was sheared using the Covaris E-Series (Covaris, Inc., Woburn, MA, USA), and the size distribution of fragments was evaluated using Agilent 2200 TapeStation (Agilent Technologies, Inc.). The DNA library was processed using the SureSelect^XT^ Target Enrichment System and captured using ClearSeq Inherited Disease^XT^, according to the manufacturer's recommendations (Agilent Technologies, Inc.). This design included 2,742 genes involved in the pathogenesis of human inherited diseases. Before the sequencing run, the captured DNA library was checked for its quality (Agilent 2200 TapeStation; Agilent Technologies, Inc.) and quantity (Qubit^®^ 1.0; Thermo Fisher Scientific, Inc.). The library was then sequenced on an Illumina MiSeq benchtop sequencer following the manufacturer's recommendations (Illumina, Inc., San Diego, CA, USA).

#### NGS data processing and data analysis

The raw sequencing data were processed using a multi-step advanced bioinformatics pipeline. The quality of the raw sequencing files was checked using FastQC (version 0.11.5; http://www.bioinformatics.babraham.ac.uk/projects/fastqc/). The presence of adapters was scanned using minion and swan (Kraken package, version 15-065) (12). The preprocessing of raw sequencing files was performed using Cutadapt (version 1.11) (13). Briefly, very low-quality ends were trimmed (Phred<5). Then, the adapters from both reads of a pair were removed with a minimal overlap of 3 bp and a maximum of 10% mismatch in a matched sequence (removed adapters: R1, AGATCGGAAGAGCACACGTCTGAACTCCAGTCAC; R2, AGATCGGAAGAGCGTCGTGTAGGGAAAGAGTGTAGATCTCGGTGGTCGCCGTATCATT). Finally, very short (<35 bp) and unpaired reads after the trimming were discarded. Preprocessed sequencing reads were mapped to a reference genome (hg19; University of California Santa Cruz Genome FTP; http://hgdownload.cse.ucsc.edu/goldenPath/hg19/chromosomes/) (14,15) by BWA aln/sampe (version 0.7.15) (16). Alignments were further processed with Stampy (version 1.0.29) (17). Aligned bam files were sorted by position and mate information was corrected using Samtools (version 1.3) (18). Since the library was sequenced in two separate sequencing runs, bam files were merged into one using Picard (version 2.1.0; http://picard.sourceforge.net). PCR duplicates were marked and removed using Picard. Duplicate-clean bam files were indel realigned using GenomeAnalysisTK (version 3.6) (19). Base Quality Score Recalibration (BQSR) was performed in two steps using GenomeAnalysisTK, and dbSNP (version 147) variants were used as a set of known variants (https://www.ncbi.nlm.nih.gov/snp). The coverage of targeted regions was explored using bedtools (version 2.23.0) (20). Additional quality checks and statistics were obtained using Picard.

Raw variant calls were performed using VarScan2 (version 2.4.2) (21) with default settings, except that the minimal variant frequency was set to 0.2, and the VarScan2 P-value set to 0.05. The dbSNP ID was added if a match between dbSNP and raw variants was found using SnpSift (version 4.2) (22). Filtering of the raw variants was performed using VarScan2 with the default settings, except for the minimal P-value, which was set to 0.05. SNPs in very close proximity to indels were removed from the calls. The effect of the variants, ClinVar (downloaded on 10/18/2016) (23) and dbSNP annotation was added to the filtered variant calls using SnpEff (version 4.2) (24). SnpEff also provided putative variant impacts (HIGH, MODERATE, LOW, MODIFIER) to categorize and prioritize variants.

Final variants were extracted from the filtered variants by targeted regions and by association with genes of interest using vcftools (version 0.1.15) and bcftools (version 1.3) (25). Additional analyses and annotations were performed using R (version 3.3.1; http://www.r-project.org/) with the data.table (version 1.9.6; http://CRAN.R-project.org/package=data.table) and VariantAnnotation (26) libraries. The NGS data were manually checked and visualized using Integrative Genomics Viewer (version 2.3.82) (27,28). NGS data in .fastq and .bam format are available in the Array Express database (https://www.ebi.ac.uk/arrayexpress/) under the acces-sion number E-MTAB-7026.

The variants were classified using ACMG recommendations (29) and detailed information provided in databases OMIM (30), ClinVar (23), dbSNP (https://www.ncbi.nlm.nih.gov/snp), UniProtKB/Swiss-Prot (31), ExAc (32), 1000 Genomes (33) and relevant scientific literature. The web-based application gene.iobio 3.0.5 (http://gene.iobio.io/) was used to assess the variant's localization within the context of known pathogenic or likely pathogenic variants from ClinVar (23). The *in silico* analysis was performed using online tools: PROVEAN Tool (34), Polyphen-2 (35), MutationTaster2 (36) and VarSome (37).

#### Sanger sequencing and data analysis

The pathogenic or likely pathogenic single-nucleotide variants (SNVs) in the proband and parents were validated using targeted Sanger sequencing. DNA primers were designed using Primer3 (38,39), Primer Blast (40), UCSC *In-Silico* PCR (15) and OligoAnalyzer 3.1 tools (https://eu.idtdna.com/calc/analyzer), and were synthesized by Integrated DNA Technologies, Inc. (Coralville, IA, USA). PCR was performed using the forward primer 5′-CAGAGCAACACAGCTTTGGA-3′ and a reverse primer 5′-GGAGACATTTCCAGGCCCTAT-3′, according to the manufacturer's recommendations (Promega Corporation). PCR products were purified using the Exonuclease I and FastAP Thermosensitive Alkaline Phosphatase protocol (Thermo Fisher Scientific, Inc.). Then, single-stranded DNA fragment libraries for direct Sanger sequencing were prepared using the BigDye^®^ Terminator v3.1 Cycle Sequencing kit (Thermo Fisher Scientific, Inc.), according to the manufacturer's recommendations. The sequencing reactions were run on the DNA sequencer ABI 3130 (Applied Biosystems; Thermo Fisher Scientific, Inc.). The analysis was performed using Sequencher^®^ version 5.1 software (http://www.genecodes.com; Gene Codes Corporation, Ann Arbor, MI, USA). The chromatograms of the proband and the parents are stored in the Figshare online digital repository (doi: 10.6084/m9.figshare.6744224).

## Results

### 

#### Whole-genomic analyses and verification

The proband was assessed to have a normal female karyotype (46, XX) via cytogenetic analysis. Consequently, the array-CGH analysis of the oligonucleotide DNA microarray 180 K CGH exhibited the same result [arr(1–22,X)×2]. Due to the unexplained severe pathological phenotype, which was suspected to have a molecular genetic cause, the proband and her parents were involved in a pilot project using a targeted NGS approach with capture design ClearSeq Inherited disease (performed on proband DNA samples), with consecutive Sanger sequencing verification (performed on proband and parental DNA samples). In the course of the NGS data analysis, the primary focus of the study was exonic variants, and the data were filtered for non-synonymous exonic variants (SNVs and indels) only. These variants were analyzed in detail by searching through the ClinVar, OMIM, dbSNP and UniProtKB/Swiss-Prot databases. Then, *in silico* tools were used to predict the structural and functional impact of these variants on the encoded protein (PROVEAN Tool, Polyphen-2, MutationTaster2 and VarSome). Finally, the findings were correlated to the patient phenotype, and were verified by independent analysis of Sanger sequencing in the proband and unaffected parents.

**Figure 1.**
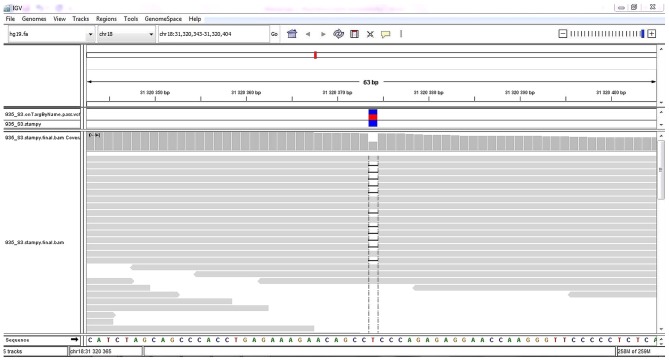
Graphical report of the 1 bp deletion variant NC_000018.9:g.31320374delT in the *ASXL3* gene detected by the targeted next-generation sequencing. The 1 bp deletion is represented by a gap in the particular reads. This variant is present in 47.92% (23/48) of the proband's reads in this region. It leads to the frameshift protein variant p.R1004Efs*21 characterized by the occurrence of a premature termination codon.

Quality control of mapped reads to genomic targets was performed. A total of >99% of reads were mapped to genomic targets, with 30X coverage for >90% of bases. In the proband, a total of 18,558 DNA sequence variants in targeted regions were identified, including 17,560 variants (94.7%) classified with known SNP identification numbers. In total, 16,794 SNVs, 887 insertions and 877 deletions were identified. By performing NGS data analysis and variant filtering, a heterozygous 1-bp deletion variant NC_000018.9:g.31320374delT (NM_030632.1:c.3006delT) affecting the *ASXL3* gene was identified, and it was present in 47.92% of reads (23/48) covering this position (Fig. 1). No other pathogenic or likely pathogenic variants related to the phenotype of the proband were identified. This variant was not found in the ExAC or 1000 Genomes databases. An *in silico* prediction analysis was performed using the MutationTaster2 and VarSome tools to identify its impact on the ASXL3 protein structure and function. This single-nucleotide deletion in the *ASXL3* gene results in a p.R1004Efs*21 frameshift leading to a premature termination codon. The protein structure is likely to be affected due to the strong protein truncation (>50% of protein length is missing). Using the VarSome prediction tool, the p.R1004Efs*21 frameshift was classified as likely pathogenic following the ACMG criteria (29) (Fig. S1).

**Figure 2.**
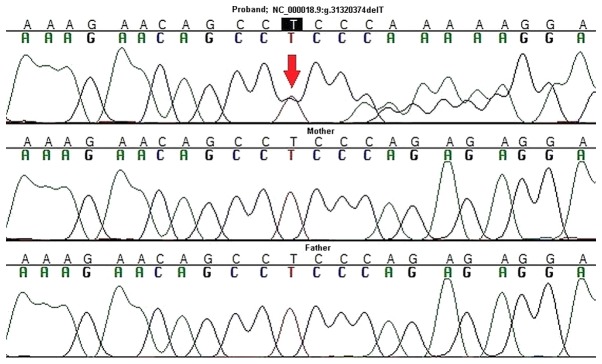
Validation and parental analysis of the variant NC_000018.9:g.31320374delT in the *ASXL3* gene by Sanger sequencing. The *de novo* origin and heterozygous state of the variant in the proband (red arrow) was validated. The upper chromatogram is that of the proband, while the middle and bottom chromatograms are those of the mother and father, respectively.

Consequently, targeted Sanger sequencing of DNA from the proband and her parents was performed to validate the *ASXL3* variant and to assess its origin (*de novo*, or paternal/maternal). A *de novo* origin and heterozygous state was confirmed for p.R1004Efs*21 using this method (Fig. 2). This result provides strong supporting evidence for its pathogenic or likely pathogenic effect.

Using gene.iobio.io 3.0.5, it was determined that the proband p.R1004Efs*21 variant is located in the same cluster as other known pathogenic and likely pathogenic *ASXL3* variants, which enhances its clinical relevance (Fig. S2).

## Discussion

The present study reports the case of a child with severe psychomotor delay, hypotonia, microcephaly and facial dimorphism. Using targeted NGS and following verification analysis by Sanger sequencing, a novel *de novo* pathogenic sequence variant in the *ASXL3* gene, p.R1004Efs*21, was identified.

The *ASXL3* gene is located at the 18q12.1 chromosome region and encodes a member of the vertebrate ASX-like protein family. ASXL family members represent epigenetic scaffolding proteins that assemble specific epigenetic regulators and transcription factors to specific genomic loci containing histone modifications (41,42). Polycomb protein ASX (ASX) was first identified in *Drosophila melanogaster* as a part of the polycomb group of proteins involved in embryonic development, maintaining HOX genes in a transcriptionally repressive state (43,44). The encoded protein ASXL3 contains 12 exons and has 2,248 amino acids. Like other ASX family members, the ASXL3 protein has a conserved domain structure: ASXN and ASXH domains in the N terminus; ASXM1 and ASXM2 domains in the middle region; and the PHD domain in the C terminus (41,42). The ASXN and PHD zinc-finger domains play a role in the regulation of gene transcription, representing putative DNA- or histone-recognition sites. The region around the ASXH domain creates protein-protein interaction sites for association with epigenetic regulators. The ASXM1 and ASXM2 domains are involved in protein-protein interactions. Between ASXH and ASXM1, there is the 5′ mutational cluster region (MCR), where truncated or splice variants tend to cluster, giving rise to aberrant ASXL proteins with intact ASXN and ASXH domains, while pathogenic sequence variants in the 3′MCR lead to variants with changes between the ASXM1 and ASXM2 domains (45).

Recent studies have highlighted important roles for the *ASXL3* gene in congenital disorders (45) and cancer (40) through the identification of multiple *ASXL3* pathogenic sequence SNVs and indels. Focusing on congenital disorders, the pathogenic variants affecting the *ASXL3* gene have been identified as the cause of disorders in patients sharing similar phenotypic features, including delayed psychomotor development with missed milestones, microcephaly, severe persisting feeding difficulties, poor growth, poor or absent speech and dimorphic facial features (4,5,6). As presented, the proband exhibits most of the typical BRS-related phenotypic features (abnormal pregnancy, preterm birth and reduced birth parameters, feeding difficulties, craniofacial abnormalities, psychomotor delay and autistic features). Brain MRI scans in patients with BRS usually indicate mild white matter loss and mild corpus callosum hypoplasia, or mild cerebellar vermis hypoplasia. With the exception of the brain MRI findings, these features are different to those previously described as a consequence of pathogenic variants in the *ASXL1* gene, causing Bohring-Opitz syndrome (BOS) (46). A recent study identified a group of six unrelated patients having *de novo* heterozygous truncating variants in the *ASXL2* gene (47). These patients shared specific clinical features, some of which are also present in patients with the *ASXL1* gene (BOS) and *ASXL3* gene (BRS) truncating or splicing pathogenic variants (developmental and intellectual impairment, facial dimorphism, feeding difficulties in the neonatal period and hypotonia). Since 2013, ~30 patients with *ASXL3* pathogenic truncating or splicing pathogenic variants causing BRS have been identified in scientific publications, to the best of our knowledge. The variants are clustered predominantly in two previously reported separated MCRs in exon 11 and exon 12, respectively (45).

In the proband, a novel truncating variant, p.R1004Efs*21, located in the terminal part of exon 11 outside the reported MCRs, was identified. The mutation is located in an amino acid coding region conserved among vertebrates; thus, the MutationTaster2 and VarSome tools predicted a deleterious effect of this variant on protein structure and function due to truncation. Moreover, as mRNAs containing a premature stop codon can undergo degradation through nonsense-mediated decay (NMD) (4), the truncating variants may have a loss-of-function (LoF) impact. Functional studies have suggested that all *ASXL3* nonsense variants may be translated to prematurely terminated proteins, which may consequently act in a dominant-negative way (4,5). The correlation between the position of the truncating variant and the severity of the phenotype has also been debated: The disease severity may decrease as the variant occurs further away from the 5′-end of exon 11 and towards the 3′-end (5). A recently described case of a male child with atypical BRS also revealed the occurrence of a novel heterozygous *de novo* variant, p.P1010Lfs*14, in the *ASXL3* gene (48). This variant leads to an identically truncated ASXL3 protein as that seen in the proband of the present study, but the phenotypic features between the cases are different, namely the absence of apparent structural brain abnormalities and less prominent facial dimorphism in the proband.

As an exceptional case of two compound heterozygous variants in the *ASXL3* gene, there was a reported case of another patient harboring a missense variant, p.R989G, of maternal origin, and a missense variant, p.K1026N, of paternal origin (49). The patient experienced BRS-like features and primary insulin-like growth factor 1 deficiency. That study predicted that the phenotype was the product of the synergistic or additive effect of these missense variants in the LoF of the *ASXL3* gene. The *ASXL3* gene has also been previously identified as an ASD risk gene, particularly missense variants of this gene (50). In the ClinVar database (23), there are no reported cases of patients with a BRS phenotype having heterozygous *de novo* pathogenic missense variants in the *ASXL3* gene.

Unfortunately, BRS is not a common clearly recognizable syndromes, especially due to the absence of specific phenotypic features and the low total number of reported cases. Among patients with BRS, it is possible to observe varying degrees of phenotypic severity, especially varying in the degree of ID (5). This range in severity may have an early or late post-zygotic origin, leading to pathogenic variants with somatic mosaicism, which will then have an incomplete penetrance and/or variable phenotypic features (5). Two of the described variants occur at the 3′-end of the gene (p.E1761fs, p. E1824K), indicating escape from NMD and the retention of protein activity. These observations also confirm that a broad allelic heterogeneity may be implicated in the pathogenesis of BRS.

Based on previously reported cases, the penetrance of truncated or spliced pathogenic variants seems to be high, and these have a *de novo* origin since they are absent in healthy individuals. In the ExAC database (32) there are four reported LoF variants (three exonic truncating variants and one splicing variant), none of which have an allelic frequency higher than 8.34×10^−6^ (exonic truncating variants) or 1.38×10^−5^ (splicing variant), and the probability of LoF intolerance is estimated to be 1.00. This value predicts high intolerance of the gene to LoF mutations (32). A total of >670 missense variants have been reported at present, and their allele frequency varies significantly (from 8.281×10^−6^ to 0.6029), occurring across the whole coding region of the *ASLX3* gene. This observation is indicative of the different impact of missense variants on the ASXL3 protein function, from benign polymorphisms to possibly damaging effects (which was evaluated using Polyphen-2 *in silico* prediction) (35).

In the present study, a novel *de novo* frameshift variant in the *ASXL3* gene, which could lead to a truncated protein in a young patient with severe psychomotor developmental delays and microcephaly, was detected. Based on these findings and previous reported cases, it was proposed that the identified frameshift variant in the *ASXL3* gene may have significantly affected ASXL3 protein structure and function. These observations may help in defining and diagnosing BRS in patients with similar phenotypic features.

## Supplementary Material

Supporting Data

## Figures and Tables

**Table I.** Clinical features of the proband and summary of selected previous patients with the additional sex-combs like 3 pathogenic variants.

								Craniofacial features			Muscular abnormalities										
																
Author, year	Sex (F/M ratio)	Pregnancy (normal/abnormal ratio)	Birth (weeks of gestation)	Birth OFC (cm)	Birth weight (kg)	Birth length (cm)	Feeding difficulties	Strabismus	Palate abnormalities	Microcephaly	Hypotonia	Hypertonia	Motor delay	Free walking	Intellectual disability	Speech impairment	Autistic features	Brain MRI (normal/abnormal ratio)	EEG (normal/abnormal ratio)	Skeletal abnormalities	(Refs.)
Present study	F	AN	34	34.5	2.16	45	yes	yes	yes	yes	yes	no	yes	no	yes	yes	yes	N	N	no	
Kuechler *et al*, 2017	3/3	2/3	36–39	32-37.5	2.66–3.6	46–52	6/6	5/6	5/6	1/6	6/6	2/6	6/6	2/6	6/6	6/6	5/6	2/4	3/3	6/6	6
Bainbridge	2/2	1/3	38–40	reduced	small	small	3/4	n.r.	3/4	3/4	3/4	1/4	4/4	no	4/4	3/4	0/4	0/1	n.r,	3/4	5
*et al*, 2013				3/4	size 3/4	size 3/4															
Balasubramanian *et al*, 2017	6/6	11/1	34-N	n.r.	m. red.	n.r.	9/12	7/12	9/12	7/12	11/12	0/12	m. del.	m. del.	11/12	11/12	9/12	9/3	n.r.	7/12	45
Srivastava *et al*, 2016	1/2	2/1	N	n.r.	1/3 reduced	n.r.	3/3	0/3	n.r.	0/3	3/3	0/3	3/3	0/3	3/3	3/3	n.r.	1/2	2/1	1/3	4

F, female; M, male; N, normal; AN, abnormal; OFC, occipitofrontal circumference; MRI, magnetic resonance imaging; EEG, electroencephalography, n.r., not reported; m. red., mostly reduced; m. del., mostly delayed.

## Data Availability

The microarray data analyzed in the present study are available in the Array Express database (https://www.ebi.ac.uk/arrayexpress/) in the .txt format under the accession number E-MTAB-7027. The NGS data analyzed in this study are available in the Array Express database (https://www.ebi.ac.uk/arrayexpress/) in .fastq and .bam format under the accession number E-MTAB-7026. Sanger sequencing data, including the chromatograms of the proband and the parents, are stored in the Figshare online digital repository (doi: 10.6084/m9.figshare.6744224).
