# Supplementary material for: Novel de novo frameshift variant in the ASXL3 gene in a child with microcephaly and global developmental delay
Source: Mol Med Rep. 2019 May 27;20(1):505–12. doi: 10.3892/mmr.2019.10303 (PMC6579994; doi:10.3892/mmr.2019.10303)

Figure S1. Output of the VarSome prediction tool for the p.R1004Efs*21 frameshift variant of the additional sex-combs like 3 gene found in the proband. The first part (‘Region browser’) shows the genomic location of the variant on chromosome 18, and the occurrence of reported variants in its proximity. The second part (‘ACMG Classification’) presents its likely pathogenic clinical impacts following the criteria of the American College of Medical Genetics. The third part (‘Transcripts’) describes the variant at the transcript level and protein level, and its location within the gene and protein.

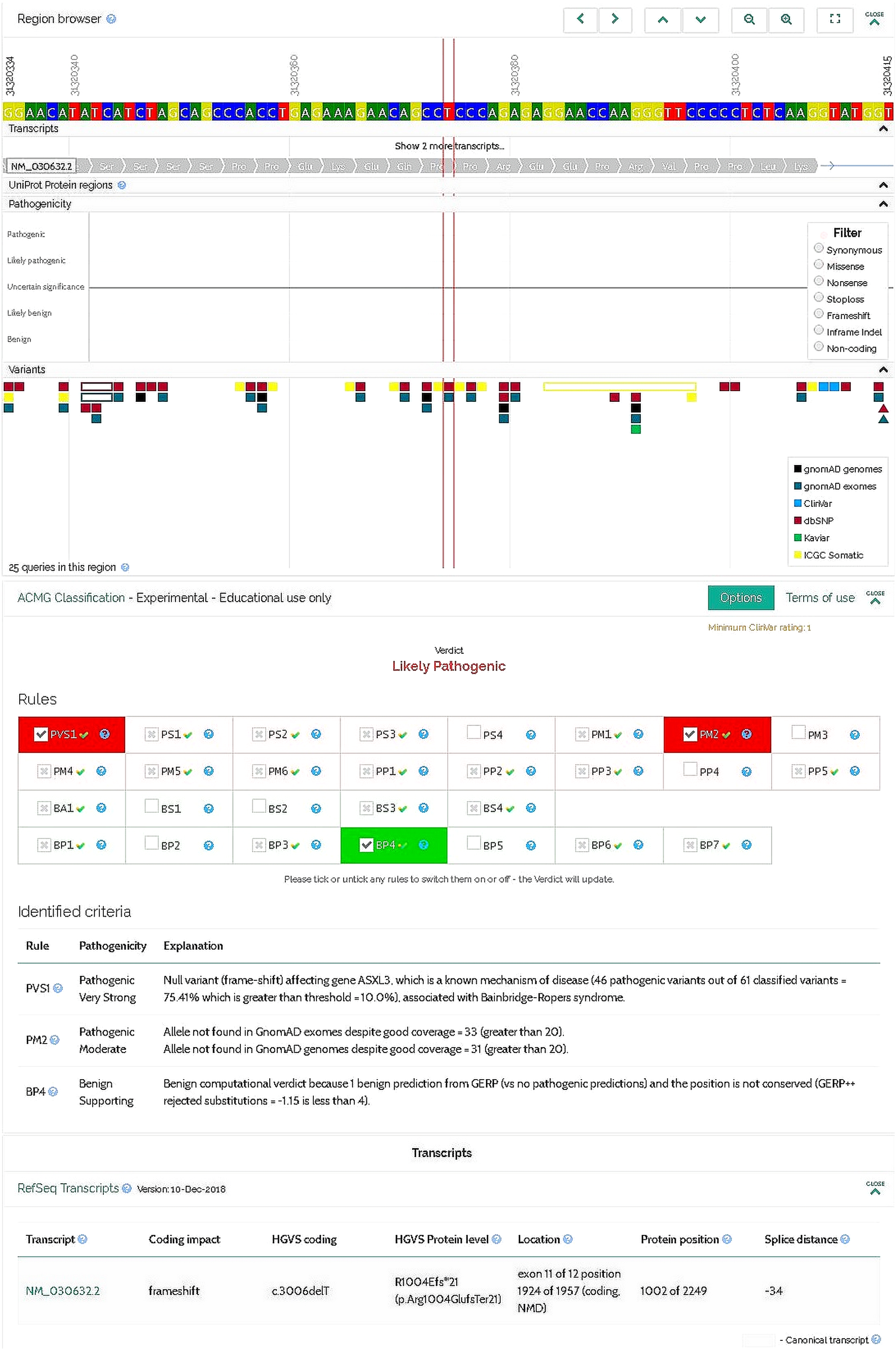

Figure S2. Output of the web-based application gene.iobio 3.0.5. for the p.R1004Efs*21 frameshift variant in the *ASXL3* gene found in the proband. The ranked variants for the *ASXL3* gene are presented in the first panel on the left side, and the flagged frameshift variant p.R1004Efs*21 is marked by the blue frame. On the right, the description of the variant, according to the nomenclature and its impact and frequency in databases, is presented. Below these two panels, the pathogenic/likely pathogenic variants of the *ASXL3* gene mentioned in ClinVar are presented. On the bottom, the *ASXL3* gene variants identified in the proband are presented, and the pathogenic frameshift variant p.R1004Efs*21 is indicated by the blue circle. The remaining *ASXL3* gene variants are classified as benign and likely benign. *ASXL3*, additional sex-combs like 3.

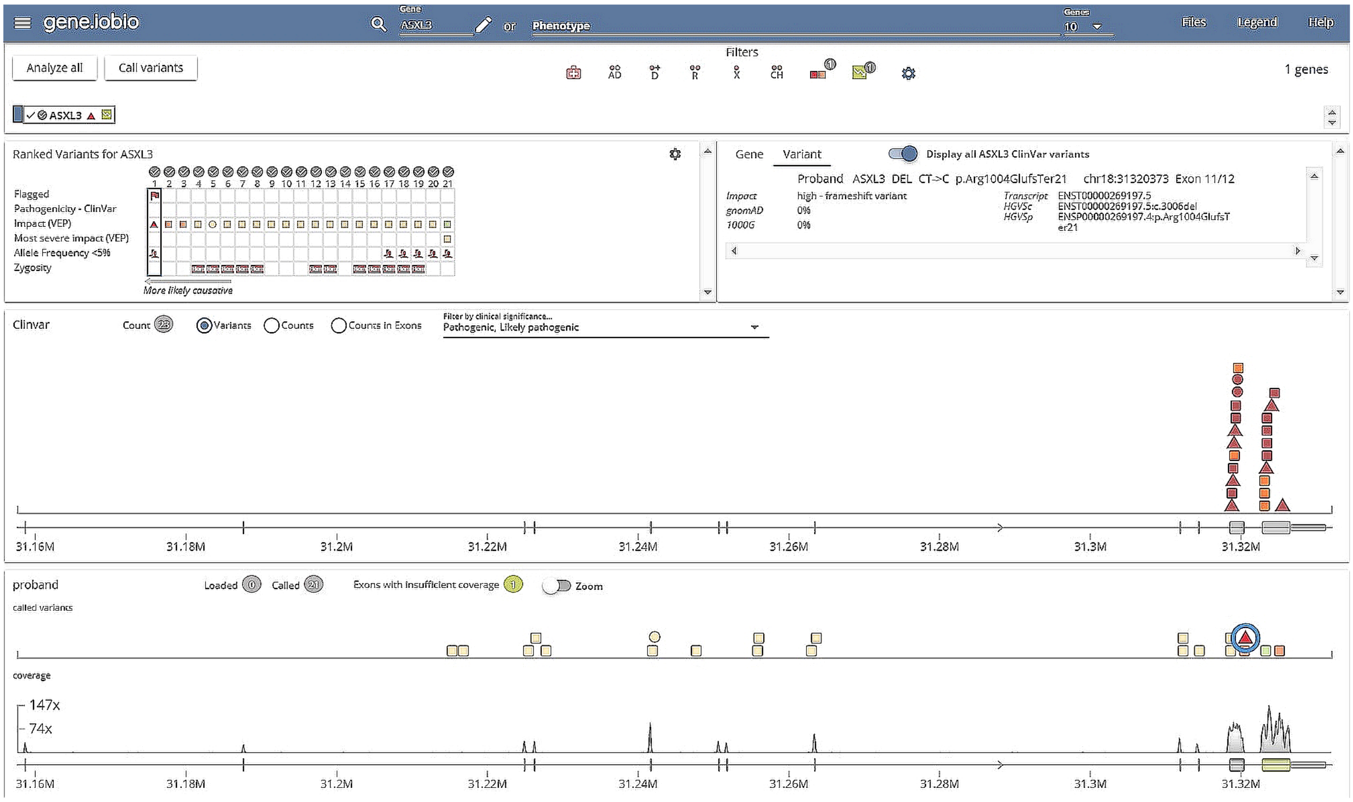

Supplement: Supporting Data [file Supplementary_Data.pdf]
